# Neoadjuvant FLOT versus SOX phase II randomized clinical trial for patients with locally advanced gastric cancer

**DOI:** 10.1038/s41467-020-19965-6

**Published:** 2020-11-30

**Authors:** Birendra Kumar Sah, Benyan Zhang, Huan Zhang, Jian Li, Fei Yuan, Tao Ma, Min Shi, Wei Xu, Zhenglun Zhu, Wentao Liu, Chao Yan, Chen Li, Bingya Liu, Min Yan, Zhenggang Zhu

**Affiliations:** 1 Department of General Surgery, Gastrointestinal Surgery Unit, Ruijin Hospital, Shanghai Jiao Tong University School of Medicine, Shanghai Key Laboratory of Gastric Neoplasms, Shanghai Institute of Digestive Surgery, Shanghai, China; 2 Department of Pathology Ruijin Hospital, Shanghai Jiao Tong University School of Medicine, Shanghai, China; 3 Department of Radiology, Ruijin Hospital, Shanghai Jiao Tong University School of Medicine, Shanghai, China; 4 Clinical Research Center, Ruijin Hospital, Shanghai Jiao Tong University School of Medicine, Shanghai, China; 5 Department of Medical Oncology Ruijin Hospital, Shanghai Jiao Tong University School of Medicine, Shanghai, China

**Keywords:** Gastric cancer, Randomized controlled trials

## Abstract

Neoadjuvant chemotherapy with docetaxel, oxaliplatin, fluorouracil, and leucovorin (FLOT regimen) has shown promising results in terms of pathological response and survival rate in patients with locally advanced resectable gastric cancer (LAGC). However, tegafur gimeracil oteracil potassium capsule (S-1) plus oxaliplatin (SOX regimen) is the preferred chemotherapy regimen in Eastern countries. Here, we conduct an open label, two-arm, phase II randomized interventional clinical trial (Dragon III; ClinicalTrials.gov: NCT03636893) to evaluate the safety and efficacy of both regimens. Patients with LAGC are randomly assigned to receive either 4 cycles of the neoadjuvant FLOT regimen (40 patients) or 3 cycles of the SOX regimen (34 patients) before gastrectomy. The primary endpoint is the comparison of complete (TRG1a) or subtotal (TRG1b) tumor regression grading in the primary tumor. There are no significant differences in adverse effects or postoperative morbidity and mortality between the two groups. No significant differences in the proportion of tumor regression grading between the FLOT group and the SOX group are found. Complete or subtotal TRG is 20.0% in the FLOT group versus 32.4% in the SOX group. Therefore, our study does not find statistically significant differences between neoadjuvant FLOT and SOX regimens for the primary outcomes reported here in locally advanced gastric cancer.

## Abstract (web summary)

Neoadjuvant FLOT regimen has shown promising results for the treatment of locally advanced gastric cancer, however SOX regimen remains the preferred chemotherapy in Eastern countries. Here the authors report that the two therapies result in similar outcomes, measured as clinical downstaging and pathological response, in a phase II randomized clinical trial.

## Introduction

For locally advanced gastric cancer (LAGC), there has been a positive trend in neoadjuvant chemotherapy after the milestone publication of the MAGIC trial in 2006, and results from this trial were recently even supported by those from clinical trials from Asian countries^1–5^. Furthermore, recent studies have shown that neoadjuvant chemotherapy is well tolerated and does not influence postoperative morbidity or mortality in gastric cancer patients^6^. A large-scale German study clearly showed the superiority of neoadjuvant docetaxel, oxaliplatin, fluorouracil, and leucovorin (the FLOT regimen) over epirubicin, cisplatin, and fluorouracil or capecitabine (the ECF or ECX regimens, respectively) in terms of pathological response and overall survival^7,8^. Docetaxel-based triplet chemotherapy, the FLOT regimen, is not a common chemotherapy in China; however, there have been published studies that show that the modified or standard FLOT regimen is safe and effective in Chinese patients^9,10^. Taxane-based triplet chemotherapy was considered more toxic in the past; therefore, doublet chemotherapy with the oral tegafur–gimeracil–oteracil potassium capsule (S-1) is the mainstream adjuvant chemotherapy in Asian countries, and a few studies have suggested S-1 plus platinum-based chemotherapy as neoadjuvant chemotherapy for locally advanced gastric cancer, especially with bulky lymph nodes^11–13^.

In recent years, several studies have been carried out in East Asian countries on the efficacy of perioperative chemotherapy for patients with LAGC. Among them, the preliminary results of two large-scale RCT trials (RESOLVE, RESONANCE) in China suggested that the neoadjuvant SOX regimen is beneficial in terms of R0 resectability, TRG, ypTNM, and pCR. Patients in the neoadjuvant chemotherapy group achieved a longer 3-year DFS than the control group^14,15^. To our knowledge, there is no previous study on neoadjuvant chemotherapy for LAGC that compared the efficacy between the SOX and FLOT regimens. For patients undergoing neoadjuvant chemotherapy, the pathological response rate or tumor regression grading is considered one of the major factors that influence overall survival^16,17^. We conducted this study to evaluate the safety and efficacy of both regimens. The main purpose of this study was to explore that whether there is a difference of pathological response after neoadjuvant chemotherapy with doublet SOX and triplet FLOT regimen.

In this work, we compare the rate of postoperative tumor regression between the neoadjuvant chemotherapy FLOT and SOX groups. Our study does not find statistically significant differences between neoadjuvant FLOT and SOX regimens for the primary outcomes reported here in locally advanced gastric cancer.

## Results

### Trial design and enrollment

**Fig. 1:**
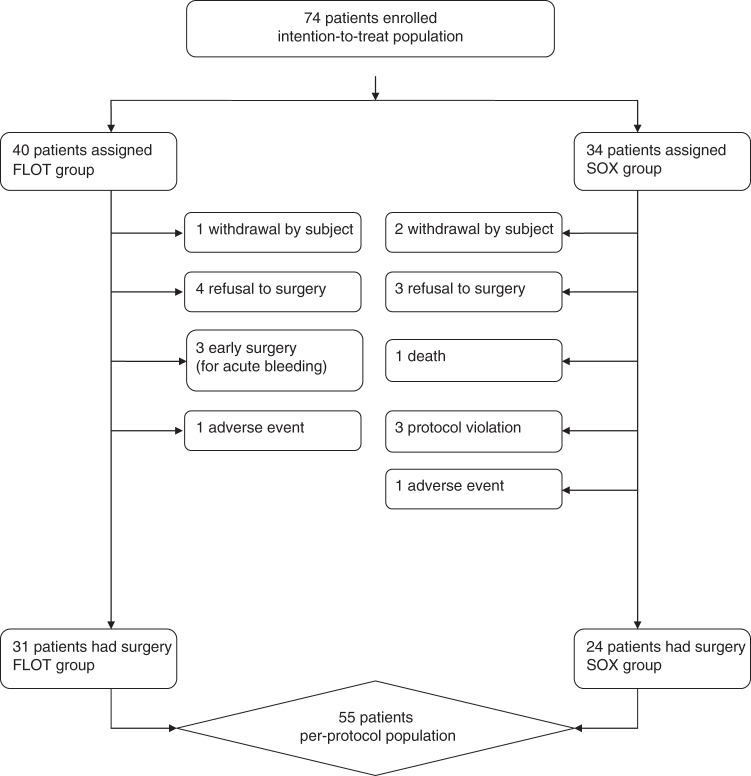
CONSORT diagram. FLOT docetaxel, oxaliplatin, fluorouracil, and leucovorin, SOX tegafur–gimeracil oteracil potassium capsule (S-1) plus oxaliplatin.

Altogether, 74 patients (40 patients in the FLOT group and 34 patients in the SOX group) were enrolled between August 2018 and March 2020. Nine patients in the FLOT group and ten patients in the SOX group dropped out for different reasons (Fig. 1). Finally, 55 patients completed the planned chemotherapy and underwent surgical resection. All cases were analyzed, no data were excluded. All 74 randomly assigned cases were considered the intention-to-treat (ITT) population, and 55 patients who had completed all planned chemotherapies and underwent surgery were considered the per-protocol (PP) population. The trial was ended after the surgical treatment of 55th patient. This was determined without any assessment of outcomes. We decided to end the trial at this point, to have a preliminary result, and discuss whether to go for phase III trial.

### Reasons for patients dropout

There was one death registered in SOX arm. The main cause of death was the grade IV hematological adverse event, followed by multiple organs failure and pulmonary infection. In the FLOT arm, one patient had acute cerebral infarction after the first chemo cycle. The patient did not continue further chemotherapy and surgical treatment. In the SOX arm, one patient was diagnosed for deep venous thrombosis. The DVT was diagnosed during evaluation for surgical treatment, i.e., 3 weeks after the completion of all three chemotherapy cycles. The patient refused to undergo surgical treatment.

There were also three cases of protocol violation in SOX arm. Two of them requested for other chemotherapies after allocation in SOX arm. One case was laparoscopically diagnosed to have peritoneal metastases after allocation, and thus did not receive neoadjuvant chemotherapy.

### Clinical characteristics

There was no significant difference in any clinical parameters between the FLOT and SOX groups, including age, sex, or BMI (Table 1, *P* > 0.05). There was no significant difference in terms of the location of the tumor or the type of resection. Thirty-two percent of patients in the FLOT group and 33.4% of patients in the SOX group had tumors in the proximal site of the stomach. All patients underwent D2 lymphadenectomy, 58.1% of patients in the FLOT group and 62.5% in the SOX group underwent total gastrectomy. One patient in each group underwent pancreatoduodenectomy due to local invasion.

**Table 1:** Demographic data.

Parameter	FLOT	SOX
Sex		
Male	29 (72.5)	21 (61.8)
Female	11 (27.5)	13 (38.2)
Age (years)		
Median	67	61
Range	27–76	34–80
Body mass index		
Median	23.41	23.18
Range	15.69–29.48	17.31–27.82
Tumor (pre-NAC CT)		
T4A	23 (57.5)	21 (61.8)
T4B	17 (42.5)	13 (38.2)
Lymph node (pre-NAC CT)		
N0	1 (2.5)	3 (8.8)
N1	4 (10.0)	6 (17.6)
N2	31 (77.5)	17 (50.0)
N3	4 (10.0)	8 (23.5)
TNM stage (pre-NAC CT)		
IIB	1 (2.5)	3 (8.8)
III	22 (55.0)	18 (52.9)
IVA	17 (42.5)	13 (38.2)

Analysis of intention-to-treat (ITT) population.

### Adverse effects

All the 55 patients in per-protocol population completed the planned chemotherapy with full dose of respective chemotherapy regime (as described in “Methods”). All patients completed three cycles of neoadjuvant chemotherapy in the SOX group and four cycles of chemotherapy in the FLOT group before surgery. There was no significant difference in chemotherapy-related hematological or nonhematological adverse effects between the two groups (Table 2, *P* > 0.05). Most of the hematological or nonhematological adverse events were below grade 3. Nine and five events of hematological grade 3–4 adverse events were observed in the FLOT and SOX groups, respectively (Table 2). Adverse events for patients who were dropped out are described in Supplementary Table 1.

**Table 2:** Adverse effects.

Parameter	FLOT	SOX
WBC decreased		
Grade 0, 1, 2	30 (96.8)	24 (100.0)
Grade 3, 4	1 (3.2)	0
Neutrophil count decreased		
Grade 0, 1, 2	29 (93.5)	22 (91.7)
Grade 3, 4	2 (6.5)	2 (8.3)
Febrile neutropenia		
Grade 0, 1, 2	31 (100.0)	23 (95.8)
Grade 3, 4	0	1 (4.2)
Anemia		
Grade 0, 1, 2	27 (87.1)	22 (91.7)
Grade 3, 4	4 (12.9)	2 (8.3)
Platelet count decreased		
Grade 0, 1, 2	30 (96.8)	24 (100.0)
Grade 3, 4	1 (3.2)	0
Aminotrasferase increased		
Grade 0, 1, 2	30 (96.8)	24 (100.0)
Grade 3, 4	1 (3.2)	0
Nausea		
Grade 0, 1, 2	30 (96.8)	24 (100.0)
Grade 3, 4	1 (3.2)	0
Vomiting		
Grade 0, 1, 2	30 (96.8)	24 (100.0)
Grade 3, 4	1 (3.2)	0
Diarrhea		
Grade 0, 1, 2	31 (100.0)	24 (100.0)
Grade 3, 4	0	0
Peripheral neuropathy		
Grade 0, 1, 2	31 (100.0)	24 (100.0)
Grade 3, 4	0	0
Fatigue		
Grade 0, 1, 2	31 (100.0)	24 (100.0)
Grade 3, 4	0	0
Anorexia		
Grade 0, 1, 2	30 (96.8)	24 (100.0)
Grade 3, 4	1 (3.2)	0
Oral mucositis		
Grade 0, 1, 2	31 (100.0)	24 (100.0)
Grade 3, 4	0	0

Analysis of per-protocol population except otherwise indicated.

### Radiological response

There was no significant difference in the pretreatment cTNM stage between the FLOT group and the SOX group (Table 1). A total of 41.9% versus 37.5% of cases were diagnosed as stage IVa in the FLOT and SOX groups, respectively. There was no significant difference concerning the radiological response rate between the two groups (Table 3). In the ITT population, the disease control rate (PR + SD) was comparable between the FLOT group (75.0%) and the SOX group (67.6%), and the overall response rate (ORR) was 55.0% in the FLOT group versus 41.2% in the SOX group (Table 3, *P* > 0.05).

**Table 3:** Post neoadjuvant chemotherapy (NAC) CT evaluation.

Parameter	FLOT	SOX	*P* value
Tumor (post-NAC CT)			
T1	1 (3.2)	1 (4.2)	0.412
T2	9 (29.0)	10 (41.7)	
T3	13 (41.9)	5 (20.8)	
T4A	8 (25.8)	8 (33.3)	
Lymph node (post-NAC CT)			
N0	4 (12.9)	7 (29.2)	0.214
N1	12 (38.7)	9 (37.5)	
N2	15 (48.4)	7 (29.2)	
N3	0	1 (4.2)	
TNM stage (post-NAC CT)			
I	3 (9.7)	5 (20.8)	0.435
IIA	7 (22.6)	6 (25.0)	
IIB	1 (3.2)	2 (8.3)	
III	20 (64.5)	11 (45.8)	
Response rate			
PR	22 (71.0)	14 (58.3)	0.691
SD	8 (25.8)	9 (37.5)	
PD	1 (3.2)	1 (4.2)	
Overall response rate (ITT population)	22 (55.0)	14 (41.2)	0.254
Disease control rate (ITT population)	30 (75.0)	23 (67.6)	0.606

Analysis of per-protocol population except otherwise indicated (the Fisher’s exact test).

### Pathological response

Among the PP population, there was no significant difference in any pathological parameters (Table 4, *P* > 0.05). Patients in both groups had favorable margin-free resection: 87.1% in the FLOT group and 100.0% in the SOX group. Lauren’s classification showed that 58.1% of tumors in the FLOT group and 54.2% of tumors in the SOX group were intestinal types. The proportion of T4a tumors and N2 lymph nodes was relatively higher in the FLOT group than in the SOX group, and a greater proportion of postoperative stage III tumors (ypTNM) was observed in the FLOT group than in the SOX group (54.8% versus 45.8%), but there was no significant difference between the two groups.

**Table 4:** Clinicopathological results of two groups.

Parameter	FLOT	SOX	*P* value
Tumor site			
Proximal	7 (22.6)	7 (29.2)	0.622
Proximal body	3 (9.7)	1 (4.2)	
Body	7 (22.6)	3 (12.5)	
Distal body	6 (19.4)	5 (20.8)	
Distal	8 (25.8)	6 (25.0)	
Total	0	2 (8.3)	
Type of gastrectomy			
Partial	13 (41.9)	9 (37.5)	0.787
Total	18 (58.1)	15 (62.5)	
Lauren’s classification			
Intestinal	18 (58.1)	13 (54.2)	0.389
Diffuse	7 (22.6)	6 (25.0)	
Mixed	3 (9.7)	5 (20.8)	
Unclassifiable	3 (9.7)	0	
Extent of resection			
R0	27 (87.1)	24 (100.0)	0.123
R1	4 (12.9)	0	
Nerve invasion			
Negative	16 (51.6)	14 (58.3)	0.785
Positive	15 (48.4)	10 (41.7)	
Vessels invasion			
Negative	25 (80.6)	17 (70.8)	0.525
Positive	6 (19.4)	7 (29.2)	
Tumor			
Unclassifiable	1 (3.2)	0	0.916
T1A	1 (3.2)	1 (4.2)	
T1B	1 (3.2)	1 (4.2)	
T2	2 (6.5)	3 (12.5)	
T3	19 (61.3)	16 (66.7)	
T4A	6 (19.4)	2 (8.3)	
T4B	1 (3.2)	1 (4.2)	
Lymph node			
N0	10 (32.3)	9 (37.5)	0.692
N1	4 (12.9)	3 (12.5)	
N2	10 (32.3)	5 (20.8)	
N3A	6 (19.4)	4 (16.7)	
N3B	1 (3.2)	3 (12.5)	
Harvested lymph nodes			
Median (no.)	34	36	0.599
YpTNM			
I	3 (9.7)	3 (12.5)	0.736
II	11 (35.5)	10 (41.7)	
III	17 (54.8)	11 (45.8)	

Analysis of per-protocol population except otherwise indicated (the Fisher’s exact test).

### Primary outcomes

The overall pathological response (TRG grade 1a + 1b + 2) rate was 67.7% in the FLOT group versus 75% in the SOX group (Table 5, *P* > 0.05). In the ITT population, the complete or subtotal TRG was 20% in the FLOT group and 32.4% in the SOX group, but there was no significant difference between the two groups (*P* > 0.05).

**Table 5:** Difference of histopathological tumor regression in intention-to-treat (ITT) population.

TRG	FLOT	95% CI	SOX	95% CI	*P* value
Complete or subtotal	8 (20.0)	10.0–39.9	11 (32.4)	17.9–58.4	0.289
1A	1 (2.5)	0.3–17.7	0	NA	1.000
1B	7 (17.5)	8.3–36.7	11 (32.4)	17.9–58.4	0.178
2	13 (32.5)	18.8–55.9	7 (20.6)	9.8–43.1	0.300
3	10 (25.0)	13.4–46.4	6 (17.6)	7.9–39.2	0.574

The Fisher’s exact test.

### Postoperative morbidity

There was no significant difference in the postoperative stay at the hospital between the FLOT group and the SOX group, and the median length of stay was 9 days in both groups. There was no significant difference in overall postoperative morbidity between the two groups (Table 6, *P* > 0.05). Two (6.5%) anastomotic leakages were observed in the FLOT group and 1 (4.2%) in the SOX group. One patient underwent reoperation for intra-abdominal hemorrhage in the SOX group. There were no deaths due to postoperative complications in either group.

**Table 6:** Postoperative complications per-protocol (PP) population.

Complication		FLOT	SOX	*P* value
Clavien Dindo grading				
Grade I		0	1	
Grade II		7	3	
Grade III	Grade IIIa	2	1	
	Grade IIIb	0	1	
Grade IV	Grade IVa	0	2	
	Grade IVb	0	0	
Grade V		0	0	
Overall complications		9 (29.0)	8 (33.3)	0.775
Abdominal complication		7 (22.6)	5 (20.8)	0.100
Intra-abdominal hemorrhage		0	1 (4.2)	0.436
Superficial wound dehiscence		0	1 (4.2)	0.436
Pulmonary infection		2 (6.5)	1 (4.2)	1.000
Abdominal infection		4 (12.9)	2 (8.3)	0.686
Anastomotic leakage		2 (6.5)	1 (4.2)	1.000
Pancreatic fistula		0	1 (4.2)	0.436
Impaired renal function		1 (3.2)	0	1.000
Cardiac/respiratory failure		0	1 (4.2)	0.436
Readmission		1 (3.2)	1 (4.2)	1.000
Reoperation		0	1 (4.2)	0.436
Death		0	0	NA

Analysis of PP population except otherwise indicated (the Fisher’s exact test).

## Discussion

Initial reports on the FLOT4 trial showed that 37% of patients in the FLOT group versus 23% in the ECF/ECX group achieved complete or subtotal tumor regression after neoadjuvant chemotherapy^7^. Further results on survival revealed that the patients in the FLOT group had an overall survival of 50 months versus 35 months in patients in the ECF/ECX group^8^. Recent small-scale studies from China also suggested that the FLOT regimen was safe and feasible in Chinese patients^9,10^. A propensity-score-matched retrospective study from China also suggested that patients with neoadjuvant FLOT had improved overall survival compared with patients who underwent surgery first^18^. The results of these studies suggested that the FLOT regimen was beneficial to locally advanced gastric cancer in terms of pathological regression and survival. However, the combination of fluorouracil and platinum chemoagents, e.g., SOX or XELOX regimens, is commonly used as neoadjuvant chemotherapy in East Asia, including Japan^11–13^. Preliminary results of two large-scale RCTs from China (RESOLVE and RESONANCE) further concluded that the SOX regimen is beneficial for LAGC^14,15^. As a result, some controversy remains regarding whether the FLOT regimen is similarly beneficial in East Asian patients. Whether there are any differences between the triplet and the doublet chemoagents in terms of adverse effects and survival benefit has not been studied. In our study, we investigated neoadjuvant FLOT and SOX regimens for patients with LAGC in an attempt to compare the adverse effects and postoperative pathological response between the two groups. Although the sample size is not large enough, to our knowledge, there was no head-to-head comparative study of the FLOT and SOX regimens as neoadjuvant chemotherapy for LAGC. The higher proportion of complete or subtotal TRG in the SOX group than in the FLOT group does not indicate the superiority of the SOX regimen over the FLOT regimen because there was no significant difference in terms of statistical calculations. However, at the very least, the results of this study urge the need for further large-scale multicenter randomized trials. Our results may provide some insights for further trials.

There was no difference in the FLOT and SOX groups in terms of adverse effects and postoperative morbidity. Thus, this study further validated the results of our previous study that neoadjuvant chemotherapy with the FLOT regimen is safe in Chinese patients^10^. We observed a very low rate of leucopenia and neutropenia in both groups. This was significantly lower than that was reported in the original FLOT trial. As per protocol, the use of GCSF was restricted to treatment only, thus preventive GCSF was not used. However, on post hoc analysis, we found GCSF was used more frequently in the FLOT group (94.9%) than in the SOX group (43.3%). The GCSF was prescribed to any patient who had WBC or Neutrophil count lower than the normal range, regardless of severity. Probably this might be the main cause of the significantly lower occurrence of grades 3–4 neutropenia in this cohort. Besides all the patients received preventive Leucogen tablets and Batilol Tablets orally, starting right after chemotherapy. Both medications are indicated for stimulating granulocytes. Therefore notably lower rate of leucopenia and neutropenia of this cohort should be interpreted considering the above factors.

In this study, the demographic data show that patients in both groups were well balanced by randomized assignment. The univariate analysis of all data suggested that TRG was associated with sex, nerve invasion, vessel invasion, and postoperative pathological stage. However, there was no difference in any of these factors between the two groups (Table 4). These data suggest that these known factors, which might have a role in a pathological response, did not influence the results of this study.

There was a conflicting result for the radiological response rate with that of pathological results, which showed that a greater proportion of ORR was seen in the FLOT group than in the SOX group, although there was no statistically significant difference. Therefore, we further analyzed the data according to the stratification of pretreatment clinical staging and postoperative pathological TNM staging. The proportion of complete or subtotal TRG was higher in the SOX group, but there was no significant difference compared to the FLOT group (Table 7).

**Table 7:** Complete or subtotal tumor regression.

Stage type	Stage	FLOT	SOX	*P* value
cTNM	IIB	1/1 (100.0)	1/3 (33.3)	NS
	III	5/17 (29.0)	6/12 (50.0)	NS
	IVA	2/13 (15.4)	4/9 (44.4)	NS
ypTNM	I	3/3 (100.0)	3/3 (100.0)	NS
	II	5/11 (45.5)	6/10 (60.0)	NS
	III	0/17	2/11 (18.2)	NS

*cTNM* pretreatment clinical TNM stage, *ypTNM* postoperative pathological TNM stage.

Analysis of per-protocol population except otherwise indicated (the Fisher’s exact test).

Approximately 37% of patients achieved complete or subtotal TRG in the FLOT4 trial^7^, but only 20% of patients achieved complete or subtotal TRG in the FLOT group of our study. Even if we did not compare the result with the SOX group, this result shows that the proportion of complete or subtotal TRG was less than the result of the FLOT4 trial. We hypothesize that the heterogeneity was caused by racial biological differences, and perhaps the FLOT regimen was not as effective in Chinese patients as it was reported to be in German patients. However, concrete data are needed to support this hypothesis. In contrast, the proportion of complete or subtotal TRG in the SOX group was 32.4%, which was comparable to the result of FLOT chemotherapy in the FLOT4 trial^7^.

We did not calculate the sample number because there was no good-quality research on tumor regression grading in the SOX regimen, and we had only the results of the FLOT4 trial. Most likely, this was the most important limitation of this study. The number of participants was empirically estimated to obtain preliminary results, and initially, 60 patients were expected to be enrolled for the analysis, but there were a substantial number of patients who dropped out of the trial. Because the primary endpoint of our study was to compare the pathological regression, the cut-off time for data analysis was set at the completion of the surgery of the 55th patient. This cut-off time was set after discussion among investigators and statisticians, without knowing the pathological results. Nonetheless, we must interpret the result of this study cautiously as the study is exploratory and therefore with no sample size calculation the power to show a difference between the arms is not demonstrated strongly. Another concern is that the primary endpoint of TRG may not translate into OS benefit therefore cannot be considered both regimens are equivalent in survival. The results of DFS and OS for this cohort are still awaited, which will provide further insight into these findings. Of course, further multicenter RCT studies are necessary to elaborate the differences between the FLOT regimen and the SOX regimen as neoadjuvant chemotherapy for patients with LAGC in terms of overall survival.

Our study did not find statistically significant difference between neoadjuvant FLOT and SOX regimens for locally advanced gastric cancer patients in terms of clinical downstaging and pathological response. There was no significant difference in adverse effects and postoperative morbidity between the two groups. The results for disease-free survival and overall survival are still awaited. A large-scale phase III multicenter randomized controlled trial is necessary for the validation of this result.

## Methods

This was an investigator-initiated, phase II, open-label, randomized controlled trial. The first patient was enrolled on August 22, 2018, and the last patient was enrolled on November 14, 2019. Data of all patients were collected between August 2018 and March 2020 at Ruijin Hospital, Shanghai Jiao Tong University School of Medicine, a large volume dedicated center for gastric cancer in Shanghai, China. An English translation of the main sections (including study design, inclusion/exclusion criteria, and pre-specified outcomes) of the original study protocol is available as Supplementary Note 1 within the Supplementary Information file. This trial is registered at ClinicalTrials.gov with trial number: NCT03636893 (Dragon III: Neoadjuvant Chemotherapy (FLOT Versus SOX) for Gastric Cancer).

### Inclusion criteria

Inclusion criteria were set as patients with any sex, age between 18 and 80 years old, a non-obstructive tumor of the stomach or esophagogastric junction, histologically confirmed adenocarcinoma with clinical-stage within cTNM: cT3 to cT4b, lymph node N1–N3 and without distant metastases (M0). Other inclusion criteria were adequate hematological function, liver function, renal function, heart function, and pulmonary function. Performance status was required to have below 2 according to Eastern Cooperative Oncology Group (ECOG). Written informed consent from the patient was mandatory for enrollment.

### Exclusion criteria

Patients were excluded if they were confirmed or highly suspicious for distant metastases, retroperitoneal lymph node metastases, locally invaded irresectable tumors, recurrent gastric cancer, secondary malignant disease, had prior chemo- or radiotherapy, or participated in another clinical trial. Patients were also excluded if they had acute infectious diseases, uncontrolled systemic disease or comorbidities, known contraindications, or hypersensitivity to chemotherapeutic agents.

### Reasons for dropout

After enrollment in the study, the patients were dropped out if they did not comply with the study protocol or withdrew the consent for any reason. Patients were also dropped out if they were unable to complete planned treatment for any reason, refused to undergo surgery at the same hospital.

### Ethics

The study was performed according to the Declaration of Helsinki and Good Clinical Practice Guidelines as defined by the International Conference on Harmonization. The institutional review board of Ruijin Hospital approved the study protocol, and patients gave written informed consent for the planned treatment. The study was conducted and analyzed by Unit III of the Department of Gastrointestinal Surgery at Ruijin Hospital. This study was monitored by the Clinical Research Center of Ruijin Hospital (the official body responsible for guiding and monitoring all types of research in the hospital), and a timely meeting was performed to check the implementation of protocol guidelines.

### Pretreatment assessment

All patients completed all the routine tests, including, but not limited to, the following:

1. Complete blood count, liver and renal function test, clotting analysis, serum tumor biomarkers.
2. Electrocardiography, echocardiography, plain chest radiography.
3. Upper gastrointestinal endoscopy and biopsy for pathological diagnosis.
4. Enhanced computed tomography of the chest, abdomen, and pelvis. CT examination included arterial, venous, and portal phases. CT images consisted of transverse, sagittal, and coronary sections.
5. For suspicious distant metastases, supraclavicular lymph nodes or retroperitoneal lymph nodes on CT, we performed ultrasound tests or magnetic resonance imaging (MRI) as appropriate.
6. Patients underwent bone scintigraphy or positron emission tomography (PET) for suspicious lesions on CT examination.
7. Finally, the diagnosis of peritoneal metastases was made by exploratory laparoscopy.
8. For clinical staging of the disease, we followed the eighth edition of the tumor–node–metastasis (TNM) classification, issued by the International Union against Cancer (UICC).

### Recruitment

Any gastric cancer patient who was admitted at the center, and met the criteria for inclusion was considered for recruitment, thus we confirm that there was no selection bias during recruitment. Patients were screened by the trained clinicians at the designated center and the principal investigators were responsible for the evaluation of pretreatment assessment and deciding for enrollment.

### Randomization and blinding

We applied the simple randomization without blocks and stratification of any factors. The randomization was performed using a predetermined code. The random allocation sequence was generated by the statistician at the Clinical Research Center (Central body to overlook all clinical trials). The randomized code was generated by the Random Number Generators of the SPSS statistical software. And the active number generator was initialized by a reproducible fixed value. A randomized sequence was created for 1:1 allocation of 100 cases, 50 cases in each group. The random numbers were placed in sealed envelopes, and a serial number was assigned to each envelope according to the sequence of allocation of the randomized number. Each envelope was opened sequentially, according to the admission sequence of subjects.

A blinded statistician was responsible for randomized assignment of interventions, either the FLOT or the SOX group. The assignment was made by telephone contact or text messages after the patient met the inclusion criteria and signed the informed consent form. The patient and care givers were not masked after allocation of intervention. Outcome assessment for the primary endpoint was performed by strictly blinded pathologists. Other data for post hoc analysis were also collected by blinded clinicians.

### Neoadjuvant chemotherapy

Patients were transferred to the Department of Medical Oncology for chemotherapy, a standard protocol for chemotherapy was circulated, and timely inspection was performed by the investigators and members of the Clinical Research Center to evaluate the implementation of the protocol. Antiemetic drugs with dexamethasone were routinely administered intravenously before chemotherapy. Other supportive drugs, including granulocyte colony-stimulating factor (GCSF), were given for treatment purposes only. Preventive use of GCSF was not allowed. Surgical intervention was allowed for an emergency, e.g., acute upper gastrointestinal bleeding or perforation.

Patients in the FLOT group received four cycles of standard FLOT chemotherapy^7^, and the patients in the SOX group received three cycles of S-1 plus oxaliplatin before curative gastrectomy.

A cycle of FLOT chemotherapy consists of the following:

Day 1: Intravenous 5-fluorouracil (5-FU) 2600 mg/m² via peripherally inserted central catheter (PICC) continued for 24 h

Intravenous leucovorin 200 mg/m^2^

Intravenous oxaliplatin 85 mg/m^2^

Intravenous docetaxel 50 mg/m^2^

The next chemotherapy cycle was repeated on the 15th day.

The cycle of SOX chemotherapy consisted of the following:

Day 1: Intravenous oxaliplatin 130 mg/m²

Day 1–14: Oral tegafur–gimeracil oteracil potassium capsule (S-1) 80 mg/m² twice/day.

The next chemotherapy was repeated on the 22nd day.

### Evaluation of adverse effects

Adverse effects were recorded according to the National Cancer Institute Common Terminology Criteria for Adverse Events (CTCAE 4.0). Drug dose or timing was adjusted for patients with grade three and above adverse effects. Patients with progressive disease were allowed to have alterations in their treatment. Patients were allowed to withdraw from the study for any reason.

### Tumor restaging

Radiologists followed the guidelines of Response Evaluation Criteria in Solid Tumors (RECIST version 1.1) for comparison of radiological response to neoadjuvant chemotherapy^19^. Two specialized radiologists independently evaluated the response rate, and the final result was obtained after reviewing both results.

### Surgery

Patients underwent surgical resection between 2 and 4 weeks after the completion of neoadjuvant chemotherapy. Exploratory laparoscopy was routinely performed to rule out peritoneal or distant metastases. Only specialist surgeons for gastric cancer were allowed to perform the surgery, and all surgeons were fully aware of the study protocol. Partial or total gastrectomy with D2 lymphadenectomy was performed according to Japanese gastric cancer treatment guidelines^19,20^. Patients with adjacent involved organs underwent combined resection along with gastrectomy. Combined resection was allowed only if R0 resection could be achieved. Distal gastrectomy with Billroth I gastroduodenostomy or Billroth II gastrojejunostomy with Braun anastomosis or Uncut Roux-en-Y gastrojejunostomy or Roux-en-Y gastrojejunostomy was performed for the tumors located at the antrum or lower part of the stomach body. Total gastrectomy with Roux-en-Y esophagojejunostomy was performed for the proximal or large tumors at the body of the stomach. The extent of the surgery was documented to state whether the procedure was curative or noncurative according to the definition stated in Japanese gastric cancer treatment guidelines^20^.

### Pathological assessment

Pathologists were blinded to the allocation types of neoadjuvant chemotherapies. After formalin fixation and paraffin embedding, pathologists performed an immunohistochemical examination of all resected specimens. The routine examination included the tumor type; the depth of tumor invasion; the involved lymph nodes; the resection margins; and the invasion of nerves, lymphatics, or blood vessels. Resection or R status was nominated for curative resection (R0) or noncurative resection (R1 and R2). Pathological examinations also included the following: the measurement of the macroscopically identifiable residual tumor and/or scarring indicating the site of the previous tumor bed. Two specialized pathologists followed the Becker criteria for tumor regression grading (TRG)^16^. Any conflicting results were settled after re-examination and discussion among both pathologists and investigators.

### Tumor regression grade (TRG) and Becker criteria

Tumor regression grade according to Becker criteria included “Grade 1a” (Complete tumor regression i.e., 0% residual tumor per tumor bed), “Grade 1b” (Subtotal tumor regression i.e., <10% residual tumor per tumor bed) “Grade 2” (Partial tumor regression i.e., 10–50% residual tumor per tumor bed), “Grade 3” (Minimal or no tumor regression i.e., >50% residual tumor per tumor bed).

### Primary outcomes

Total percentage of patients with pathologically complete tumor regression (TRG1a) and subtotal tumor regression (TRG1b) in the primary tumor; blinded pathologists assessed the TRG after having specimen of the last enrolled patient.

### Secondary outcomes

Overall survival (OS) and disease-free survival (DFS) were set as the secondary endpoints, which will be assessed after five years from the surgery of the last enrolled patient. OS and DFS were defined as the time from randomization to death from any cause and the time from randomization to relapse of disease, respectively.

### Sample size and power calculation

This was an exploratory study. Sample was not estimated according to statistical power calculation; the sample size was estimated empirically. The data analysis cut-off time was set at the completion of surgery for the 55th patient, who met the criteria for per-protocol (PP) analysis.

### Statistics and reproducibility

The primary endpoint was analyzed in the ITT population, defined as all the patients who were randomly assigned to a treatment. Postoperative morbidity and mortality were analyzed in the PP population, which is the number of patients who had surgery after the completion of all planned neoadjuvant chemotherapy. Comparisons of other factors except primary endpoint were post hoc analyses. The statistical analysis was performed with Statistical Package for Social Science (SPSS) version 22.0 for Windows (SPSS, Inc., Chicago, IL). Kolmogorov–Smirnov Test was used to test the normality of the data. The continuous data are described as the median and range. Categorical data are expressed as frequencies and percentage. The Fisher’s exact test was used to compare the differences in rate between the two groups. Univariate analysis for the association of TRG with clinicopathological factors was performed with Fisher’s exact test. An exploratory analysis with Fisher’s exact test was done to compare the differences in rate of TRG according to pretreatment clinical staging and postoperative pathological TNM staging. All *P* values presented are two-sided, and a *P* value < 0.05 was considered statistically significant. All the described results were tested for replication by two independent statisticians. Each statistician checked the results twice, thus altogether the results were successfully replicated for four times.

### Reporting summary

Further information on research design is available in the Nature Research Reporting Summary linked to this article.

## Supplementary information

Supplementary Information

Reporting summary

## Data Availability

All the original clinical data can be made available on reasonable request from the corresponding author (Birendra Kumar Sah) at any time in a de-identified manner. A translated copy of the main sections of the original study protocol is available in the Supplementary Information file. The remaining data are available within the Article, Supplementary Information, or available from the authors upon request.
