# Supplementary material for: Neoadjuvant FLOT versus SOX phase II randomized clinical trial for patients with locally advanced gastric cancer
Source: Nat Commun. 2020 Nov 30;11:6093. doi: 10.1038/s41467-020-19965-6 (PMC7705676; doi:10.1038/s41467-020-19965-6)
Supplement: Supplementary file 1 — Supplementary Information [file 41467_2020_19965_MOESM1_ESM.pdf]

Supplementary Table 1 Adverse events in dropped out cases

Parameter		FLOT	SOX
Did not receive chemotherapy		1	4
Death		0	1
WBC decreased	Grade 0,1,2	8	5
	Grade 3,4	0	1
Neutrophil count decreased	Grade 0,1,2	8	5
	Grade 3,4	0	1
Febrile neutropenia	Grade 0,1,2	8	5
	Grade 3,4	0	1
Anemia	Grade 0,1,2	6	4
	Grade 3,4	2	2
Platelet count decreased	Grade 0,1,2	8	5
	Grade 3,4	0	1
Aminotrasferase increased	Grade 0,1,2	7	5
	Grade 3,4	1	1
Nausea	Grade 0,1,2	8	6
	Grade 3,4	0	0
Vomiting	Grade 0,1,2	8	6
	Grade 3,4	0	0
Diarrhea	Grade 0,1,2	8	6
	Grade 3,4	0	0
Peripheral neuropathy	Grade 0,1,2	8	6
	Grade 3,4	0	0
Fatigue	Grade 0,1,2	8	6
	Grade 3,4	0	0
Anorexia	Grade 0,1,2	8	6
	Grade 3,4	0	0
Oral mucositis	Grade 0,1,2	8	6
	Grade 3,4	0	0

Supplementary Note 1

Brief translation of the study protocol

Document update date 2017-1-10

Title

DRAGON III- Phase 2 research:

Neoadjuvant Chemotherapy (FLOT versus SOX) for Gastric Cancer

Purpose

To compare the safety and efficacy of two main streams of chemotherapy (FLOT Versus SOX) for locally advanced gastric cancer patients in a high volume center of China. To obtain preliminary results for designing a new phase III multicenter randomized controlled trial.

Nature of the study

Investigator-initiated, phase II, open-label, randomized controlled study.

Sample size

This is an exploratory study. The sample size is estimated empirically. Initially, 60 patients are anticipated to complete the planned chemotherapy and surgery. The enrollment of patients is not limited to 60 patients. The principal-investigator can decide on the study termination time or the number of patients after discussion among investigators.

Randomization

Patients will be randomized as 1:1 proportion to FLOT or SOX group. A masked statistician at the Clinical Research Center of the hospital is responsible for randomization. The assignment will be made by telephone contact or text messages after the patient met the inclusion criteria and signed the informed consent.

Endpoints

<u>Primary Outcome Measure</u>

Total percentage of patients with pathological complete tumor regression (TRG1a) and sub-total tumor regression (TRG1b) in the primary tumor

<u>Secondary Outcome Measures:</u>

Overall survival (OS): Time from randomization to death from any cause

Disease-free survival (DFS): Time from randomization to relapse of the disease

Study design

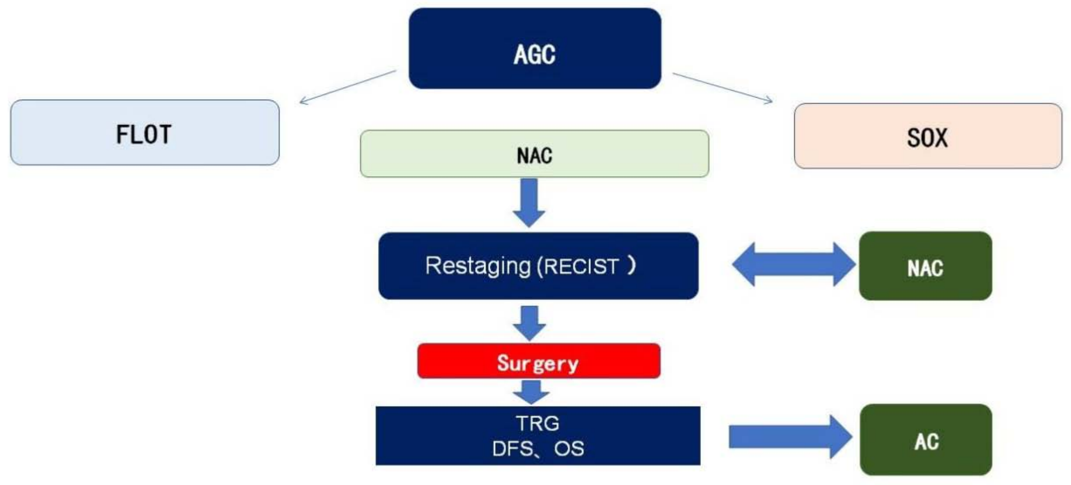

NAC: Neoadjuvant chemotherapy

AC: Adjuvant chemotherapy

RECIST: Response Evaluation Criteria in Solid Tumors

TRG: Tumour regression grade

Pre-treatment assessment

All patients will undergo a full clinical assessment before commencement of the treatment, which includes a full medical history, physical examination, a complete blood count, clotting analysis, serum liver function, and renal function test, 24-hour urinary clearance, blood tumor markers for gastrointestinal diseases. Electrocardiography, echocardiography, chest radiography, computed tomography (CT) of the chest, abdomen, and pelvis, upper gastrointestinal endoscopy are mandatory. Specially designed protocol for CT examination will be used for clinical staging of gastric cancer, which consists of arterial, venous, and portal phase of transverse section images and reconstruction images of the sagittal and coronary section). Ultrasonography, Magnetic resonance (MR) are recommended if clinically necessary to rule out suspicious distant metastases or retroperitoneal lymph nodes. Positron emission tomography (PET) or whole-body bone scintigraphy is recommended in suspected cases. Diagnostic laparoscopy is recommended to rule out peritoneal metastases. Clinical staging will be performed according to the International Union against Cancer (UICC), Tumor–Node–Metastasis (TNM) Classification of Stomach, eighth edition.

Neoadjuvant Chemotherapy

Patients will be transferred to the department of medical oncology for chemotherapy, the standard protocol for chemotherapy will be circulated and timely inspection will be performed by investigators and Clinical Research Center to evaluate the implementation of the protocol. Preventive antiemetic and dexamethasone are allowed before chemotherapy, growth factor, or other supportive medicines are allowed for treatment only. Surgical intervention is allowed for acute bleeding or other surgical emergencies.

Patients in the FLOT group will receive four cycles of the standard dose of FLOT chemotherapy and the patients in the SOX group receives three cycles of the standard dose of TGO plus oxaliplatin before undergoing curative gastrectomy.

Inclusion criteria

Age: 18-80 years old

Sex: all

Histology confirmed non-obstructive adenocarcinoma of the stomach or esophagogastric junction.

Clinical stage: cTNM: stage III or above

Performance status: Eastern Cooperative Oncology Group ECOG $\leq$2 (normal to symptomatic but in bed less than half the day)

Clinically fit for systemic chemotherapy and gastric cancer surgery, i.e. adequate renal, hepatic, hematologic, and pulmonary function.

Written informed consent

Exclusion criteria

Clinically unfit for systemic chemotherapy and gastric cancer surgery, i.e. uncontrolled cardiac disease, or other clinically significant uncontrolled comorbidities, unable to undergo general anesthesia

Confirmed distant metastases

Locally advanced inoperable disease (Clinical assessment)

Relapse of gastric cancer

Malignant secondary disease

Prior chemo or radiotherapy

Inclusion in another clinical trial

Known contraindications or hypersensitivity for planned chemotherapy

Drop out

Protocol violation

Unable to complete planned chemotherapy or surgery for any reason

Refusal to perform surgery at the same hospital

Withdrawn by the participant for any reason

Neoadjuvant Treatment

<u>FLOT Chemotherapy regimen</u>

A cycle consists of

Day 1: 5-FU 2600mg/M2 intravenous

Via peripherally inserted central catheter (PICC) for 24 hour

Day 1: Leucovorin 200mg/M2 intravenous

Day 1: Oxaliplatin 85mg/ M2 intravenous

Day 1: Docetaxel 50mg/M2 intravenous

Repeated every 15th day

SOX Chemotherapy regimen

A cycle consists of

Day 1: Oxaliplatin 130mg/M2 intravenous

Day 1-14 Tegafur gimeracil oteracil potassium capsule 80mg/M2 oral (twice daily)

Repeated every 21st day

Restaging

Response to treatment will be evaluated according to the Response Evaluation Criteria in Solid Tumors (RECIST version 1.1) guidelines. Complete Response (CR): Disappearance of all target lesions. Any pathological lymph nodes (whether target or non-target) must have a reduction in short axis to <10 mm. Partial Response (PR): At least a 30% decrease in the sum of diameters of target lesions, taking as reference the baseline sum diameters. Progressive Disease (PD): At least a 20% increase in the sum of diameters of target lesions, taking as reference the smallest sum on study (this includes the baseline sum if that is the smallest on the study). In addition to the relative increase of 20%, the sum must also demonstrate an absolute increase of at least 5 mm. (Note: the appearance of one or more new lesions is also considered progression). Stable Disease (SD): Neither sufficient shrinkage to qualify for PR nor sufficient increase to qualify for PD, taking as reference the smallest sum diameters

while on the study. The overall response rate (ORR) is defined as the percentage of patients who have achieved CR and PR. Disease Control Rate (DCR) is defined as the percentage of patients who have achieved CR, PR, and SD. Two specialized radiologists will independently evaluate the ORR and DCR. Any conflicting results will be settled after discussion among both radiologists and investigators. The principal-investigator has the right to decide on the final result.

Surgery

Surgery is recommended for two to four weeks after completion of the planned chemotherapy. The patient will undergo an exploratory laparoscopic examination to rule out peritoneal or distant metastases. Surgery will be terminated if there is peritoneal or distant metastasis.

Surgeons who are trained for gastric cancer surgery are allowed to perform the surgery. Surgeons will be trained by principal investigators on the implementation of protocol guidelines. Japanese gastric cancer treatment guidelines will be followed for surgical treatment. Surgical extent is determined by the operating surgeons during surgery, standard gastrectomy with curative intent (partial or total gastrectomy with D2 lymphadenectomy) is recommended surgical procedure. It involves resection of at least two-thirds of the stomach with a D2 lymph node dissection. Gastrectomy with combined resection of adjacent involved organs is allowed if required for R0 resection.

Resection margin: A sufficient resection margin is required when determining the resection line in gastrectomy. The proximal margin of at least 3 cm for non-infiltrative

and 5 cm for those with infiltrative tumors are recommended. Distal gastrectomy with gastroduodenostomy or gastrojejunostomy is recommended for the tumors located at the antrum or lower part of the stomach body. Total gastrectomy with Roux-en-Y esophagojejunostomy is recommended for the proximal tumors. Surgeons are required to document whether the procedure is curative or non-curative intent according to the definition stated in Japanese gastric cancer treatment guidelines. Non-curative surgery is allowed for clinical treatment but those cases will be dropped out for pathological analysis.

Pathological assessment

After formalin fixation and paraffin embedding, all specimens will have an immunohistochemical examination. Pathological assessment will be made according to local clinical guidelines that follow Tumor–Node–Metastasis (TNM) Classification of Stomach, eighth edition. Assessments include tumor type, Lauren's classification, depth of invasion, the involvement of lymph nodes, resection margins, vessels, and nerve invasion. The presence or absence of residual tumor after surgery is described as the R status; R0 is a curative resection with negative resection margins; R1 andR2 is considered non-curative resections. The tumor regression grading (TRG), Becker criteria will be used for the evaluation of pathological response in the resected specimens. Pathologists will examine residual vital tumor cells and the remnant of the previous tumor as necrosis, fibrosis, or scar as suggested in Becker criteria for grading. Two specialized pathologists will independently rate the TRG grading. Any conflicting results are settled after re-examination and discussion among both

pathologists and investigators. The principal-investigator has the right to decide on the final result.

Tumor regression grade (TRG), Becker criteria

Grade 1a: Complete tumor regression: 0% residual tumor per tumor bed

Grade 1b: Subtotal tumor regression: <10% residual tumor per tumor bed

Grade 2: Partial tumor regression: 10-50% residual tumor per tumor bed

Grade 3: Minimal or no tumor regression: >50% residual tumor per tumor bed

Statistical plan

General principle: Apply the standard statistical methods to analyze the following parameters in both arms

Timeline for the first and last patient enrolled

Total number of enrolled cases in two arms

Eligible cases: Total number of enrolled cases minus drop out cases

Comparison of basic demographic data (age, sex, BMI, etc)

Duration of chemotherapy, no. of cycles (non-parametric)

Reasons for therapy discontinuation

Resection rates

Types of surgery (surgical extent)

Postoperative morbidity and mortality

Overall Response rate (ORR): Radiological assessment (Recist 1.1)

Pathological response rate (Tumor regression grading, TRG)

Overall survival (OS)

Relapse free survival (RFS)

Availability of data

All the data will be kept safe for at least two years after the completion of the research and can be provided to any reasonable request according to local rules and regulations.
