# Supplementary material for: Neoadjuvant FLOT versus SOX phase II randomized clinical trial for patients with locally advanced gastric cancer
Source: Nat Commun. 2020 Nov 30;11:6093. doi: 10.1038/s41467-020-19965-6 (PMC7705676; doi:10.1038/s41467-020-19965-6)
Supplement: Supplementary file 2 — Reporting summary [file 41467_2020_19965_MOESM2_ESM.pdf]

nature research

Corresponding author(s): BIRENDRA KUMAR SAH

Last updated by author(s): Nov 7, 2020

Reporting Summary

Nature Research wishes to improve the reproducibility of the work that we publish. This form provides structure for consistency and transparency in reporting. For further information on Nature Research policies, see our Editorial Policies and the Editorial Policy Checklist.

Statistics

For all statistical analyses, confirm that the following items are present in the figure legend, table legend, main text, or Methods section.

n/a	Confirmed	
☐	☒	The exact sample size (n) for each experimental group/condition, given as a discrete number and unit of measurement
☒	☐	A statement on whether measurements were taken from distinct samples or whether the same sample was measured repeatedly
☐	☒	The statistical test(s) used AND whether they are one- or two-sided<br>*Only common tests should be described solely by name; describe more complex techniques in the Methods section.*
☐	☒	A description of all covariates tested
☐	☒	A description of any assumptions or corrections, such as tests of normality and adjustment for multiple comparisons
☒	☐	A full description of the statistical parameters including central tendency (e.g. means) or other basic estimates (e.g. regression coefficient) AND variation (e.g. standard deviation) or associated estimates of uncertainty (e.g. confidence intervals)
☐	☒	For null hypothesis testing, the test statistic (e.g. F, t, r) with confidence intervals, effect sizes, degrees of freedom and P value noted<br>*Give P values as exact values whenever suitable.*
☒	☐	For Bayesian analysis, information on the choice of priors and Markov chain Monte Carlo settings
☒	☐	For hierarchical and complex designs, identification of the appropriate level for tests and full reporting of outcomes
☒	☐	Estimates of effect sizes (e.g. Cohen's d, Pearson's r), indicating how they were calculated

Our web collection on statistics for biologists contains articles on many of the points above.

Software and code

Policy information about availability of computer code

Data collection	(SPSS,<br>RRID:SCR_002865)<br>SPSS version 22.0
Data analysis	(SPSS,<br>RRID:SCR_002865)<br>SPSS version 22.0

For manuscripts utilizing custom algorithms or software that are central to the research but not yet described in published literature, software must be made available to editors and reviewers. We strongly encourage code deposition in a community repository (e.g. GitHub). See the Nature Research guidelines for submitting code & software for further information.

Data

Policy information about availability of data

All manuscripts must include a data availability statement. This statement should provide the following information, where applicable:

- Accession codes, unique identifiers, or web links for publicly available datasets
- A list of figures that have associated raw data
- A description of any restrictions on data availability

All the original clinical data can be made available on reasonable request from the corresponding author at any time in a de-identified manner. A translated copy of the main sections of the original study protocol is available in the Supplementary Information file.

Field-specific reporting

Please select the one below that is the best fit for your research. If you are not sure, read the appropriate sections before making your selection.

- [x] Life sciences
- [ ] Behavioural & social sciences
- [ ] Ecological, evolutionary & environmental sciences

For a reference copy of the document with all sections, see nature.com/documents/nr-reporting-summary-flat.pdf

Life sciences study design

All studies must disclose on these points even when the disclosure is negative.

Sample size	This was an exploratory study. Sample was not estimated according to statistical power calculation.<br>The sample size was estimated empirically. The data analysis cut-off time was set at the completion of surgery for the 55th patient, who met the criteria for per-protocol (PP) analysis.<br>The trial was ended after the surgical treatment of 55th patient. This was determined without any assessment of outcomes. We decided to end the trial at this point, to have preliminary result and discuss whether to go for phase III trial.
Data exclusions	Exclusion and drop-out criteria was established in study protocol before the start of enrollment. All drop-out cases were described in CONSORT diagram and in manuscript.
Replication	All the described results were tested for replication by two independent statisticians.
Randomization	We applied the simple randomization without blocks and stratification of any factors. The randomization was performed using a predetermined code. The random allocation sequence was generated by the statistician at the Clinical Research Center (Central body to overlook all clinical trials). The randomized code was generated by the Random Number Generators of the SPSS statistical software. And the active number generator was initialized by a reproducible fixed value. A randomized sequence was created for 1:1 allocation of 100 cases, 50 cases in each group. The random numbers were placed in sealed envelopes, and a serial number was assigned to each envelope according to the sequence of allocation of the randomized number. Each envelope was opened sequentially, according to the admission sequence of subjects.
Blinding	A blinded statistician was responsible for randomized assignment of interventions, either the FLOT or the SOX group. The assignment was made by telephone contact or text messages after the patient met the inclusion criteria and signed the informed consent form. The patient and care givers were not masked after allocation of intervention. Outcome assessment for the primary endpoint was performed by strictly blinded pathologists. Other data for post hoc analysis were also collected by blinded clinicians.

Reporting for specific materials, systems and methods

We require information from authors about some types of materials, experimental systems and methods used in many studies. Here, indicate whether each material, system or method listed is relevant to your study. If you are not sure if a list item applies to your research, read the appropriate section before selecting a response.

Materials & experimental systems

n/a	Involved in the study	
x		Antibodies
x		Eukaryotic cell lines
x		Palaeontology and archaeology
x		Animals and other organisms
	x	Human research participants
	x	Clinical data
x		Dual use research of concern

Methods

n/a	Involved in the study	
x		ChIP-seq
x		Flow cytometry
x		MRI-based neuroimaging

Human research participants

Policy information about studies involving human research participants

Population characteristics	Basic characteristics: Sex, age, BMI,TNM stage.<br>Pre Chemotherapy TNM stage, Post Chemotherapy TNM stage<br>Pathological Tumor regression grading (TRG)for SOX<br>TRG for FLOT
Recruitment	Any gastric cancer patient who was admitted at the center, and met the criteria for inclusion was considered for recruitment, thus we confirm that there was no selection bias during recruitment. Patients were screened by the trained clinicians at the designated center and the principal investigators were responsible for the evaluation of pretreatment assessment and deciding for enrollment.
Ethics oversight	institutional review board (IRB), Ruijin Hospital, and Clinical Research Center(Ruijin Hospital)

Note that full information on the approval of the study protocol must also be provided in the manuscript.

Clinical data

Policy information about clinical studies

All manuscripts should comply with the ICMJEguidelines for publication of clinical research and a completedCONSORT checklist must be included with all submissions.

Clinical trial registration	ClinicalTrials.gov (number NCT03636893)
Study protocol	Full study protocol can be accessed on ClinicalTrials.gov
Data collection	All data were prospectively collected between August 2018 and March 2020 at Ruijin Hospital, Shanghai Jiaotong University School of Medicine, a large volume dedicated center for gastric cancer in Shanghai, China.
Outcomes	Primary endpoints<br>Total percentage of patients with pathologically complete tumor regression (TRG1a) and subtotal tumor regression (TRG1b) in the primary tumor<br>The primary endpoint was analyzed in the intention-to-treat (ITT) population, defined as all the patients who were randomly assigned to a treatment. Postoperative morbidity and mortality were analyzed in the per-protocol (PP) population, which is the number of patients who had surgery after the completion of all planned neoadjuvant chemotherapy. Comparisons of other factors except primary endpoint were post hoc analyses.<br><br>Secondary endpoints<br>Overall survival (OS): Time from randomization to death from any cause<br>Disease free survival (DFS): Time from randomization to relapse of disease<br>Secondary endpoints will be assessed after five years from the surgery of the last enrolled patient.
